# Surgery followed by 585 nm pulsed‐dye laser therapy in the treatment of granuloma faciale

**DOI:** 10.1002/ski2.230

**Published:** 2023-03-19

**Authors:** Linh Ha‐Wissel, Lasse Kröger, Ewan A. Langan, Birgit Kahle, Katharina Boch

**Affiliations:** ^1^ Department of Dermatology University of Lübeck Lübeck Germany; ^2^ Comprehensive Center for Inflammation Medicine University‐Hospital Schleswig‐Holstein Lübeck Germany; ^3^ Manchester Sciences University of Manchester Manchester UK

## Abstract

Granuloma faciale is a rare, benign skin disease characterised by solitary or multiple papules, plaques or nodules, most often occurring on the face. The skin disorder is often associated with exacerbations and remissions; spontaneous resolution seldom occurs. The treatment of granuloma faciale is challenging. Various topical and systemic treatments, but also surgical and laser therapies have been administered. A spatially confined thermal destruction of the tissue is achieved by strong absorption of haemoglobin at 585 nm wavelength. Of note, none of the presently available therapeutic interventions are particularly successful. Moreover, Granuloma faciale has the tendency to recur after treatment. Here, we present a male patient with a treatment refractory Granuloma faciale on the right cheek who was successfully treated with the combination of surgery and pulsed‐dye laser therapy. Besides the good aesthetic outcome, remission was maintained after almost 1 year of follow‐up.

## Graphical abstract

Granuloma faciale is a rare and often chronic skin disorder. The treatment of this skin condition is challenging. Here we present a case of a treatment refractory male patient, who was treated with surgery and pulse‐dye laser therapy.

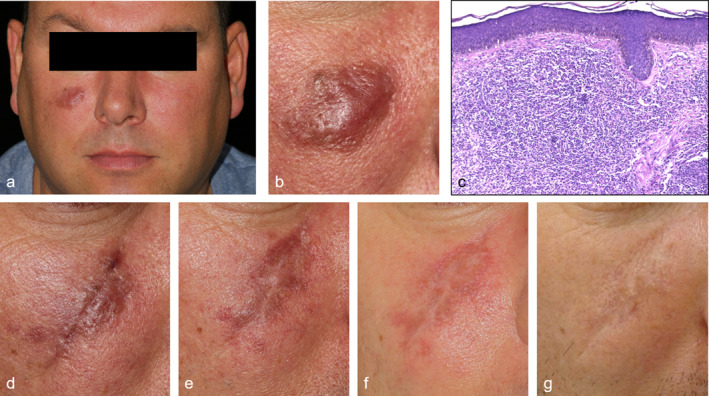

1

A 46‐year‐old Caucasian male patient was presented to our outpatient department with a 2 × 3 cm firm erythematous plaque on the right cheek (Figure 1a,b). It had been present for almost 3 years but had recently increased in size. Following histological confirmation of granuloma faciale 2 years previously, the patient had undergone treatment with dapsone for 6 months (dosage unknown) and three sessions of pulsed‐dye laser treatment (parameters unknown). Neither treatment was effective. A further biopsy was performed at the time of presentation to our department in order to confirm the diagnosis and exclude malignancy (Figure 1c). As dapsone had proved ineffective and the patient was unwilling to try topical therapy, treatment with doxycycline 200 mg q.d. Was initiated but was discontinued by the patient due to nausea and headaches after 3 weeks. Doxycycline was used off‐label, but favoured given its anti‐inflammatory and immunomodulatory effects. In view of the localisation of the skin lesions and their psychosocial impact on the patient, surgical excision was considered and a multi‐stage partial excision was performed involving two partial excisions (interval 4 months) without complications (Figure 1d,e). A complete excision was not possible due to potential disruption of the functional aesthetic unit. Hence, we decided to additionally perform 585 nm pulsed‐dye laser therapy (6 ms pulse, 8.5 J/cm^2^, 7 mm spot size). After 6 laser treatments the lesions were no longer visible (Figure 1f,g). A follow‐up after 11 months confirmed persistent.

**FIGURE 1:**
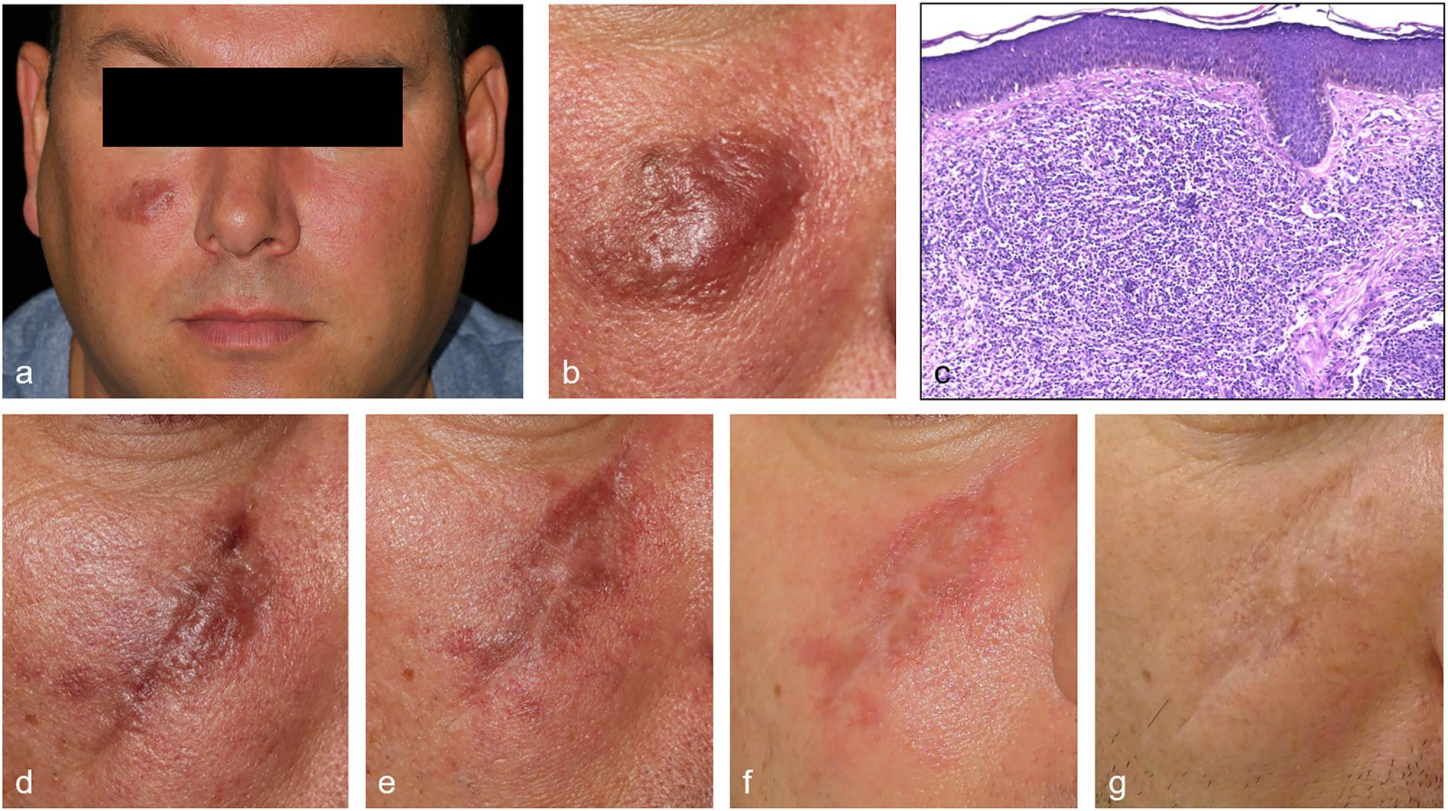
(a) Granuloma faciale on the right cheek. (b) Solitary reddish‐brown soft elevated plaque with prominent follicles. (c) Histology showing a diffuse, polymorphous inflammatory infiltrate (neutrophils, plasma cells, eosinophils, lymphocytes, and histiocytes) in the upper half of the dermis. (d) Granuloma faciale after the first partial excision. (e) Skin lesion after the second partial excision (11 months post‐operative). (f) After first laser therapy with 585 nm pulsed‐dye laser. (g) After 6 laser treatments with 585 nm pulsed‐dye laser.

Granuloma faciale is a rare, benign skin disorder characterised by solitary or multiple red‐brown, blue or violaceous papules, plaques or nodules with prominent follicles and defined borders.^1^, ^2^, ^3^ These skin lesions most often appear on the face but may rarely present on the scalp, trunk and extremities (“extrafacial granuloma faciale”).^4^ Although the lesions are usually asymptomatic, patients may report itch, pain or a stinging sensation.^1^, ^2^, ^3^ Granuloma faciale predominantly affects healthy middle‐aged white males.^2^, ^3^, ^4^ The aetiology and pathogenesis of granuloma faciale remains poorly understood, although sun exposure has been implicated as a trigger factor. Interferon‐gamma and an increased local interleukin‐5 have been suggested as potential disease mediators.^5^

A skin biopsy is necessary to confirm the diagnosis and rule out other skin diseases that have a similar clinical phenotype, including lymphoma, pseudolymphoma, cutaneous lupus erythematosus, Jessner's lymphocytic infiltration, lymphocytoma cutis, mycosis fungoides, sarcoidosis, erythema elevatum diutinum, polymorphous light eruption, insect bite reaction, or fixed drug eruption. The characteristic histological hallmarks include a mixed perivascular and interstitial infiltrate (neutrophils, lymphocytes, and plasma cells) and the presence of eosinophils in the dermis sparing the upper papillary dermis (creating a so‐called “grenz zone”). A leucocytoclastic vasculitis may be found early in the disease process, whilst an eosinophil‐rich and plasma cell‐dominant infiltrate can be seen in later stages of the disease.^6^ Direct immunofluorescence microscopy shows granular deposits of IgG, IgA, IgM and/or C3 in blood vessel walls.^7^

The treatment of this chronic skin condition is challenging. Therapeutic options include topical corticosteroids and/or calcineurin inhibitors and/or systemic immunomodulation medication (e.g. dapsone, hydroxychloroquine), but also surgical excision and laser therapy (e.g. pulsed‐dye laser, carbon dioxide laser).^8^ Based on the principle of selective photothermolysis, a spatially confined thermal destruction of the vessels is achieved by strong equal absorption of oxyhaemoglobin and deoxyhemoglobin (*µ*_*a*_ ≈ 150 cm^−1^) at 585 nm wavelength for laser pulses shorter than the thermal relaxation time τ_*r*_ = d^2^/(16κ), where *d* is the vessel diameter and *κ* is the thermal diffusivity.^9^ The estimated *τ*_*r*_ for the vessels was 7.4 ms. Of note, a sustained remission of 6 years was reported following 2 treatments of 585 nm pulsed‐dye laser using higher energy settings (450 µs pulse, 8–8.5 J/cm^2^, 5 mm spot size).^10^

The treatment of granuloma faciale remains a therapeutic challenge and is associated with significant morbidity. The combination of surgery and pulsed‐dye laser therapy might be a useful therapeutic option in treatment refractory cases.

## CONFLICTS OF INTEREST STATEMENT

Ewan A. Langan is Editor‐in‐Chief of Skin Health and Disease.

## AUTHOR CONTRIBUTIONS

**Linh Ha‐Wissel**: Investigation (Equal); Validation (Equal); Visualisation (Equal); Writing – review & editing (Equal). **Lasse Kröger**: Investigation (Equal); Visualisation (Equal); Writing – review & editing (Equal). **Ewan A. Langan**: Software (Supporting); Supervision (Equal); Writing – review & editing (Equal). **Birgit Kahle**: Conceptualisation (Lead); Investigation (Lead); Validation (Equal); Visualisation (Equal); Writing – review & editing (Equal). **Katharina Boch**: Conceptualisation (Supporting); Supervision (Lead); Visualisation (Equal); Writing – original draft (Lead).

## ETHICS STATEMENT

We confirm the patient consent for the publication of case report details and all clinical images.

## Data Availability

The data that support the findings is included in the manuscript.
